# Dynamics in the Neurotrauma Catchment Area of a German University Hospital during the COVID-19 Pandemic

**DOI:** 10.3390/healthcare10081376

**Published:** 2022-07-24

**Authors:** Rosita Rupa, Tim Vladimirov, Mirza Pojskic, Christopher Nimsky, Benjamin Voellger

**Affiliations:** Department of Neurosurgery, University Hospital Marburg, Baldingerstrasse, 35033 Marburg, Germany; rupar@med.uni-marburg.de (R.R.); tim.vladimirov@uk-gm.de (T.V.); mirza.pojskic@uk-gm.de (M.P.); nimsky@med.uni-marburg.de (C.N.)

**Keywords:** catchment area, COVID-19, neurosurgery, neurotrauma, pandemic, resource allocation

## Abstract

Objective: At the beginning of 2020, the COVID-19 pandemic enforced a rapid reallocation of healthcare resources. Our neurosurgical department is located in the German county of Marburg–Biedenkopf, about 80 km from the nearest major city. We were able to maintain our previously established open-door policy after the emergence of COVID-19. Here, we report on dynamics in the catchment area for neurotrauma patients at our department during the pandemic. Methods: 763 consecutive neurotrauma cases admitted to our department between 1 January 2018 and 31 December 2021 were analyzed retrospectively. Patients’ age, gender, origin, diagnoses, and outcomes were recorded. The number of patients hospitalized with a COVID-19 infection in Germany (PHCG) were retrieved from the Robert Koch Institute (RKI). We defined calendar weeks with >1000 PHCG as high COVID-19 caseload weeks (HCLW). Chi-square and Fisher’s exact served as statistical tests. Results: In 2020 and 2021, we observed a significantly increased number of neurotrauma patients who, with primary residence outside of our district, were admitted to our hospital compared to 2018 and 2019 (*p* < 0.001), while there were no significant differences in in-house mortality. During HCLW, a significantly increased number of neurotrauma patients with primary residence in the densely populated southwestern margin (SWM) of the contiguous part of our catchment area were referred to us compared to the time prior to the pandemic and between HCLW (*p* = 0.003). In neurotrauma patients admitted from the SWM during HCLW, there was no tendency towards higher in-house mortality. Conclusion: An open-door policy may moderate the risk of involuntarily triaging neurotrauma patients during a pandemic.

## 1. Introduction

Within the highly developed German health system [1], the running costs of hospitals are primarily funded through diagnosis-related groups (DRG)-based billing [2]. There are no predefined catchment areas, so patients may deliberately choose their doctors [3]. In the beginning of 2020, the coronavirus disease 2019 (COVID-19) pandemic, caused by severe acute respiratory syndrome coronavirus type 2 (SARS-CoV-2) [4], brought about a demand to quickly reallocate healthcare resources [5,6]. Subsequently, we and other neurosurgeons [7,8] were concerned about the possibility of an involuntary triage of neurosurgical patients due to the pandemic.

**Figure 1:**
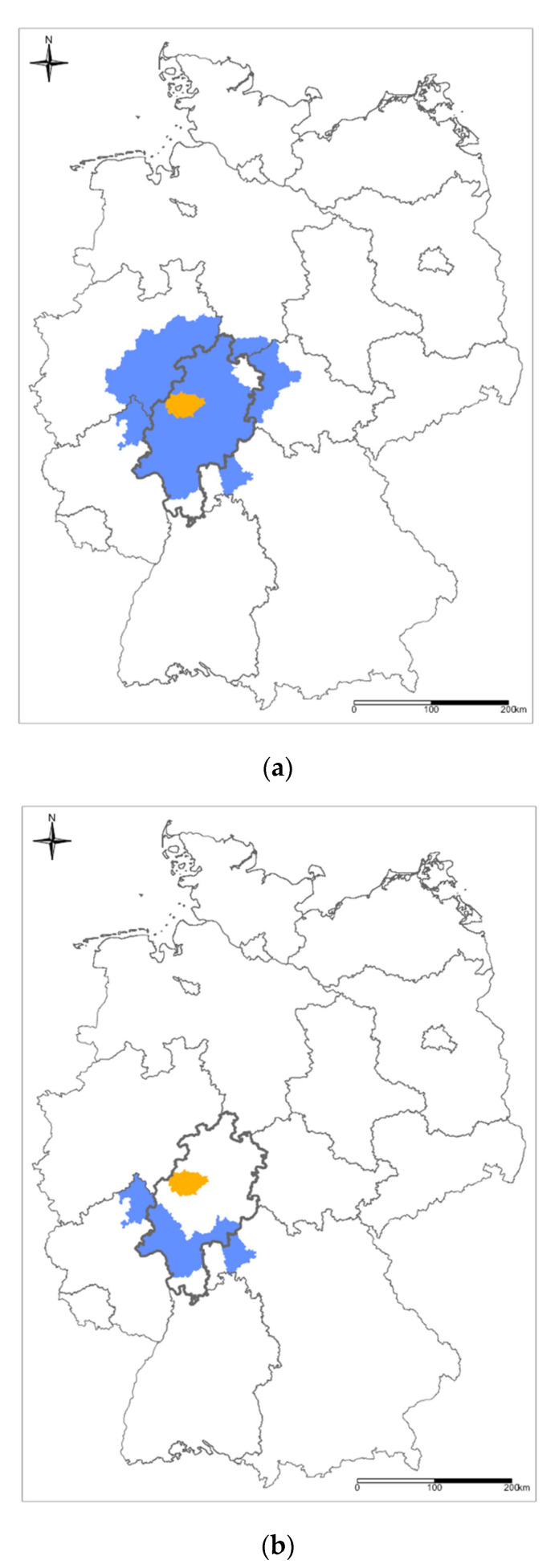
(**a**) Map of the Federal Republic of Germany. University Hospital Marburg is located in the district of Marburg–Biedenkopf (orange), which is part of the German federal state of Hesse (bold black). The contiguous part of the hospital’s catchment area for neurotrauma patients admitted between 1 January 2018 and 31 December 2021 (blue) extends into the neighboring federal states of (clockwise, beginning in the north) Lower Saxony, Thuringia, Bavaria, Rhineland–Palatinate, and North Rhine–Westphalia. (**b**) The southwestern margin (SWM; blue) of the hospital’s contiguous neurotrauma catchment area extends to the densely populated Frankfurt Rhine–-Main metropolitan region, part of which belongs to the German federal state of Hesse (bold black).

Our university hospital is located north of the Frankfurt Rhine–Main metropolitan region in the German federal state of Hesse (Figure 1), just south of a picturesque, largely pristine landscape [9]. With at least six hospitals equipped to treat neurosurgical and other emergencies around Frankfurt, and probably due to the rather remote location of our hospital, we felt the waves of the pandemic less strongly. Therefore, we were able to maintain our previously established open-door policy at our department after the emergence of COVID-19. This open-door policy basically allows any physician who encounters a neurosurgical emergency to refer the patient to us. Here, we report on dynamics in the catchment area for neurotrauma patients as observed at our department during the first two years of the COVID-19 pandemic.

## 2. Materials and Methods

A total of 763 consecutive cases admitted to our neurosurgical department between 1 January 2018 and 31 December 2021 with a main diagnosis code starting with S, according to the 10th revision of the German modification of the International Statistical Classification of Diseases and Related Health Problems (ICD-10-GM) [10], were included in this retrospective analysis. Patients with a primary residence abroad were not included. Patient data were kindly provided by our hospital’s medical controlling staff and were anonymized before processing. Table 1 provides information on patients’ age, gender, origin, main diagnoses, and outcomes.

Postal codes were converted into information based on the districts of patients’ primary places of residence. We defined the neurotrauma catchment area of our department as the entirety of districts where neurotrauma patients admitted to us between 1 January 2018 and 31 December 2021 had their primary residence. Figure 1a depicts the contiguous part of this catchment area, which extends southwards to the densely populated Frankfurt Rhine–Main metropolitan region. The southwestern margin (SWM) of the contiguous part of this catchment area comprises the districts of (in alphabetical order) Altenkirchen, Darmstadt, Frankfurt (Main), Gross–Gerau, Hochtaunus, Koblenz, Limburg–Weilburg, Main–Kinzig, Main–Spessart, Main–Taunus, Offenbach, Westerwald, and Wiesbaden (Figure 1b). To avoid excessive granularity, we did not distinguish between the city of Darmstadt and the neighboring county of Darmstadt–Dieburg, and we did not distinguish between the city of Offenbach and the neighboring county of Offenbach.

**Figure 2:**
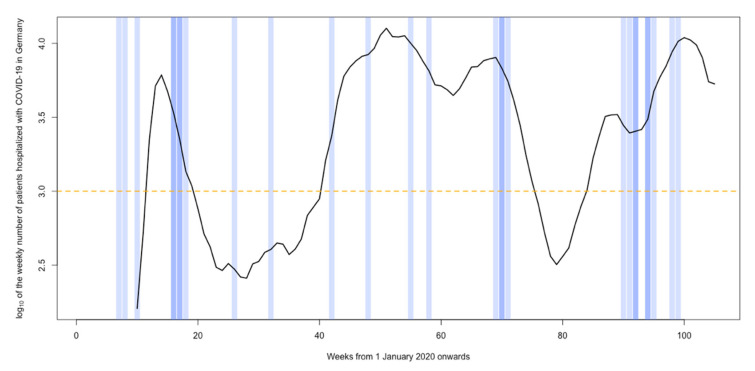
Logarithmic depiction of the weekly number of patients hospitalized with COVID-19 in Germany (PHCG) from 1 January 2020 onwards (black curve). Weeks with more than 1000 PHCG were defined as high COVID-19 caseload weeks (HCLW, black curve above the dashed orange line). Weekly numbers of neurotrauma patients admitted to University Hospital Marburg from the southwestern margin (SWM) of the hospital’s contiguous neurotrauma catchment area varied between 0 (white background), 1 (light blue background), and 2 (blue background). During HCLW, the number of neurotrauma patients admitted from the SWM was significantly higher compared to the time prior to the pandemic and between HCLW (chi-square test; *p* = 0.003).

We retrieved weekly numbers of patients hospitalized with a COVID-19 infection in Germany (PHCG), as of March 2020, online at the website of the Robert Koch Institute (RKI, [11]). Calendar weeks with >1000 PHCG, i.e., from 16 March 2020 to 10 May 2020 (weeks 12–19, 2020); from 24 August 2020 to 6 June 2021 (week 41, 2020–week 22, 2021); and from 2 August 2021 to 31 December 2021 (weeks 31–52, 2021), were defined as high COVID-19 caseload weeks (HCLW, Figure 2).

Statistics were computed and figures were created with RStudio version 2022.02.3 [12] running R version 4.0.2 [13], using geospatial data included in the geographic information system DIVA-GIS version 7.5 [14] on a macOS 12.1. Chi-square and Fisher’s exact served as statistical tests, with *p* values less than 0.05 considered statistically significant.

## 3. Results

From 2018 to 2021, we observed a slight increase in the number of districts where neurotrauma patients admitted to us had their primary residence (Table 1). At the same time, the spatial extension of the contiguous part of our neurotrauma catchment area (Figure 1a) did not change. In 2020 and 2021, significantly more neurotrauma patients who had their primary residence outside our district were admitted to our hospital compared to 2018 and 2019 (chi-square test; *p* < 0.001), while there were no significant differences in in-house mortality (chi-square test; *p* = 0.100 and above). For the respective patient numbers, see Table 2.

During HCLW, significantly more neurotrauma patients who had their primary residence in the SWM were referred to our department compared to the time prior to the pandemic and between HCLW (chi-square test; *p* = 0.003; Figure 1 and Figure 2; Table 3). For neurotrauma patients admitted from the SWM during HCLW, there was no tendency towards higher in-house mortality (Fisher’s exact test; *p* = 0.636; Table 3).

## 4. Discussion

Our spatio-temporal analysis demonstrates that continuing an open-door policy after the onset of a pandemic may allow for a significant increase in neurotrauma referrals from the margins of a hospital’s catchment area without increasing mortality. The overall small number of referrals to our department from Frankfurt Rhine–Main, selection bias prior to referral, and adequate treatment of patients may explain this observation. Our findings at least represent a strong argument in favor of continuing an open-door policy for neurotrauma patients during a pandemic, as such a policy appears useful to moderate the risk of involuntary triage.

Anywhere, however, the number of hospitalized, critically ill patients must stay below a certain threshold to allow continuing treatment of all medical emergencies. Pre-hospital triage of COVID-19 patients, e.g., at retirement homes, has been suggested to address this serious issue [15]. Looking at triage ethics from a broader perspective, one may distinguish a utilitaristic (or consequential) approach prioritizing those with the best prognosis, a deontological approach prioritizing those who are willing to be treated, an approach prioritizing those supposed to be worst off if remaining untreated, and an approach prioritizing those who have proven or are expected to fulfill certain social roles [15,16]. These potential ways to prioritize emergencies during the COVID-19 pandemic have been extensively discussed [15,17]; it has been found that subtle cultural and political nuances across Europe may result in pronounced differences as to which triage ethics are actually being recommended [15]. At our department, we base emergency treatment decisions on a patient’s prognosis and according to his/her (presumed) will.

Sufficient capacity for the treatment of any medical emergency during the pandemic requires adequate numbers of available intensive care unit (ICU) beds and staff. In response to the first wave of the pandemic, additional ICU beds were provided at our hospital and at many other hospitals throughout Germany [18]. As a result, there has never been an absolute shortage of ICU beds for neurosurgical patients at our hospital during the first year of the pandemic, despite a considerable variation in the number of regionally available ICU beds.

Our open-door policy was seriously challenged in 2021 when the easily transmissible yet highly pathogenic SARS-CoV-2 Delta variant [19] became predominant and, at the same time, the resilience of ICU nursing staff throughout Germany reached its limit [20]. As a result, closure requests in Interdisziplinärer Versorgungsnachweis (IVENA [21]), a web-based service that claims to provide real-time information on care capacity in our catchment area, were becoming increasingly common at our hospital. Services such as IVENA had been developed years ago, probably without a potential pandemic in mind. Meanwhile, experts found that the implementation of IVENA does not prevent increasing utilization of overhead resources in the emergency room [22] and leaves plenty of room to minimize the frequency of closure requests [23]. In order to decrease the risk of involuntary triage during a pandemic, we suggest that IVENA and similar services designate an adequate number of last resorts per medical specialty. Furthermore, we think communication of any given neurosurgical department’s admission policy to local authorities and to health care professionals throughout the catchment area is highly important.

In Germany, governmental regulations to ensure a minimum number of nurses per ICU bed were effective prior to the pandemic, with the intention to avoid overburdening under normal conditions [24]. At the beginning of the pandemic, the same regulations were temporarily suspended since they would otherwise have hampered access to physically available ICU beds. Although similar regulations became effective again after a few months [25], many nurses in Germany decided to terminate their employment contracts during the pandemic [20]. To address this critical development, nurses should receive adequate compensation for their work [26]. Beyond that, enhancing teamwork experience [26], preserving work-life balance [27], continuing education programs, and inhouse career opportunity offers may make the work of health care professionals more attractive. Assertive vaccination campaigns may reduce the risk of staff shortage due to SARS-CoV-2 infections during the pandemic [28].

One should bear in mind that the work presented here is a single-center retrospective analysis of what neurosurgeons at a university hospital, located away from large urban structures in Germany, experienced when treating neurotrauma patients during the first two years of the COVID-19 pandemic. The treatment of other injuries or conditions at our hospital after the onset of the pandemic was not the objective of this study. Elsewhere, circumstances might not allow neurosurgeons to maintain an open-door policy during a pandemic, with only a few suggestions left as to the further refinement of framework conditions.

## 5. Conclusions

An open-door policy may moderate the risk of involuntarily triaging neurotrauma patients during a pandemic.

## Figures and Tables

**Table 1:** Descriptive data of 763 consecutive neurotrauma cases.

Item	Year 2018	Year 2019	Year 2020	Year 2021
Total admissions (Neurosurgery)	1316	1334	1298	1187
Number of neurotrauma patients (female, male)	176 (69, 107)	194 (79, 115)	234 (101, 133)	159 (80, 79)
Median age of neurotrauma patients (min–max) in years	69 (11–95)	77 (0–97)	78 (1–98)	79 (1–100)
Districts of primary residence of neurotrauma patients	21	20	23	25
Readmissions of neurotrauma patients	2	1	0	2
Accidents at work	7	9	9	12
Main diagnoses of neurotrauma patients according to ICD-10-GM * at discharge (number of patients per diagnosis)	S06.5 (56) S06.6 (42) S06.31 (22) S02.1 (6) S12.1 (6) S06.4 (5) S02.0 (4) S06.0 (3) S06.33 (3) other (29)	S06.5 (83) S06.6 (29) S06.31 (26) S06.33 (12) S06.4 (9) S12.1 (7) S02.1 (5) S06.0 (3) S13.0 (3) other (17)	S06.5 (92) S06.6 (61) S06.21 (11) S06.31 (11) S06.33 (9) S06.4 (9) S06.0 (8) S12.1 (8) S02.0 (6) S02.1 (5) S12.24 (3) other (11)	S06.5 (58) S06.6 (26) S12.1 (9) S06.21 (7) S02.1 (5) S06.4 (5) S22.06 (5) S06.8 (4) S32.01 (4) S32.03 (4) S06.0 (3) S06.31 (3) S06.33 (3) S12.24 (3) other (20)
Neurotrauma patients deceased in hospital	11	8	24	9
Neurotrauma patients discharged to rehabilitation facility	54	75	72	56
Neurotrauma patients discharged regularly	104	106	130	90
Neurotrauma patients discharged for other reasons	7	5	8	4

* 10th revision of the German modification of the International Statistical Classification of Diseases and Related Health Problems.

**Table 2:** Primary residence, year of admission, and mortality in 763 consecutive neurotrauma cases.

Primary Residence	Years 2018, 2019	Years 2020, 2021
Marburg–Biedenkopf **	200 (8) *	152 (11) *
Elsewhere	170 (11) *	241 (22) *

* Data given as: number of patients (number of deceased patients); ** see Figure 1.

**Table 3:** Primary residence, week of admission, and mortality in 763 consecutive neurotrauma cases.

Primary Residence	HCLW **	Non-HCLW
SWM ***	22 (2) *	17 (3) *
Elsewhere	230 (21) *	494 (26) *

* Data given as: number of patients (number of deceased patients); ** HCLW: High COVID-19 caseload weeks (see Figure 2); *** SWM: Southwestern margin of the hospital’s contiguous neurotrauma catchment area (see Figure 1b).

## Data Availability

Upon reasonable request, the corresponding author will provide the anonymized dataset and R code used in this work.
